# Thymoma associated myasthenia gravis with atypical presentation of lipomatous tongue atrophy: a case report

**DOI:** 10.11604/pamj.2019.32.38.17768

**Published:** 2019-01-21

**Authors:** Suzan Salem, Islam Saad, Rana Alamri

**Affiliations:** 1 Qassim University, College of Dentistry, Department of Oral Surgery, Maxillofacial and Diagnostic Science, Kingdom of Saudi Arabia; 2 Qassim University, College of Dentistry, Periodontology and Oral Medicine, Kingdom of Saudi Arabia; 3 Saudi Board of Orthodontic, Kingdom of Saudi Arabia

**Keywords:** Thymoma, myasthenia gravis, lipomatous tongue atrophy

## Abstract

A 28-year-old female patient came to the outpatient dental clinic for multiple teeth extractions and full mouth rehabilitation suffer from myasthenia gravis (MG) primary presentation as tongue atrophy and facial muscles weakness and the symptoms became worries, the patient unable to speak as well and change her voice and complaining of dysphagia and dysarthria. Oral symptoms, treatment schedule and protocol, the selection, prescription and impacts of medications, and prevention of myasthenic crisis are all important; aspects should be considered by dentists and oral health care providers. Weakness of facial and oropharyngeal muscle is considered very popular at disease onset and therefore oral health providers are often the first medical professionals to observe these patients. Myasthenic patients seek particular approach and consultation in order to ensure ideal and proper dental management.

## Introduction

Thymus tumors, particularly lymphoepithelial thymomas, are accompanied with autoimmune pure red blood cell anemia and neuromuscular disturbances [1]. The idiom thymic epithelial tumor (TET) is more precise for describing the histogenetics of this condition. The prevalence of thymoma considered rare, with about 0.15 cases per 100,000 of the population per year [2]. Around one third of cases are found by chance upon radiographic examinations during workup for related autoimmune disorder, most commonly myasthenia gravis (MG) [3]. Thymomas in myasthenia gravis (MG) are tumors developed from thymic epithelial cells and are generally of the cortical subtype (WHO type B). Cortical thymomas commonly have some morphological resemblances with thymic cortex; both have the ability to propagate the development of immature naive CD4 T cells and export mature naive T cells into the periphery [4]. MG is a neuromuscular junction disorder manifested by muscular weakness and fatigability, owing to AChR antibodies in 85% of the cases. Thymoma MG represent about 15% of all MG cases. The immune reaction against an epitope expressed on thymoma cells spills over to neuromuscular junction components sharing the same epitope. In thymoma MG, epitopes are shared between the thymoma and muscle proteins [5, 6].

Histologically, thymomas are epithelial neoplastic cells encompassed by developing T lymphocytes. The epithelial cells have the ability to express epitopes cross-reactive with skeletal muscle proteins, such as acetylcholine receptor (AChR), titin and ryanodine receptor (RyR). The muscle-like epitopes are presented to T cells together with costimulatory molecules. Autoreactive T cells specific for AChR and titin are located in thymomas and in thymoma MG patients’ sera. Thymoma epithelial cells present AChR peptides to T-cell lines in thymoma MG patients, encouraging intrathymic immunization [7, 8]. The circulating AChR antibodies are the major reason of muscle weakness in thymoma MG, also non-AChR muscle autoantibodies interact with striated muscle titin and RyR antigens had been identified in up to 95% of MG patients with a thymoma and in 50% of late-onset MG patients. These antibodies are usually linked to more severe presentation of MG. Striational antibodies exhibited in immunofluorescence was comprised predominately from titin antibodies [9]. Disease is characterized by weakness and variability. Typically, the disease results in fluctuating weakness of striated muscle primarily affecting ocular and respiratory muscles. The disease symptoms are often magnified by the presence of physiological stress like. Diagnosis is largely based on a characteristic history of fluctuating diploma, ptosis, dysarthria and limb weakness. Dysphagia, dyspnea and head droop may also be present. The involved muscles are fatigable and symptoms improve with rest. Also masticatory action express difficulty and may develop before other symptoms giving the chance of early diagnosis and treatment of the disease [10].

Thymoma MG can be a challenging circumstances for dentists, oral health care providers and furthermore to the patients themselves. Indeed, several essentials features of thymoma MG may significantly effect on the dental treatment of these patients. Hence, dental specialists are advantageous to detect early signs of trimethylglycine (TMG), particularly those involving the head, neck and oro-pharyngeal sites and can significantly benefit patients by being familiar with this disease [10]. We report here an atypical case of thymoma associated MG with lipomatous atrophy of the tongue with a complete absence of other myasthenic signs and symptoms.

## Patient and observation

**Figure 1:**
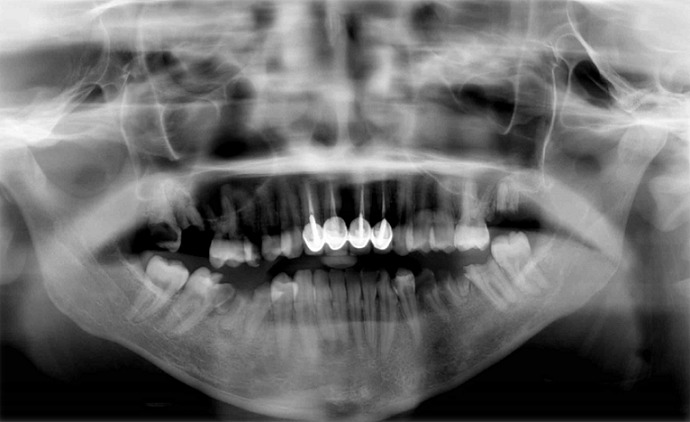
digital panoramic radiograph reveals multiple fixed prosthodontic restorations and multiple carious teeth, multiple remaining roots

**Figure 2:**
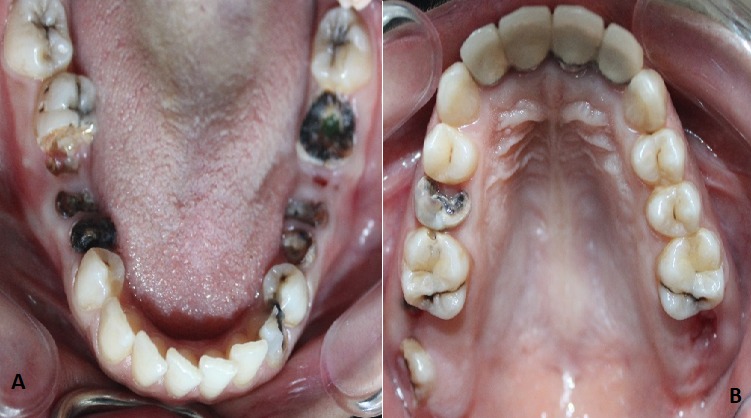
(A, B) clinical picture reveals multiple fixed prosthodontic restorations and multiple carious teeth, multiple remaining roots

A 28-year-old female patient presented to outpatient dental clinics for full mouth rehabilitation. On physical examination, the patient was awake, alert, oriented, and her blood pressure, temperature and heart rate were within normal limits. Upon taking medical history the patient revealed that was diagnosed with thymoma MG and on medication including pyridostigmine bromide 60 mg, azathioprine 50 mg and prednisone 20 mg. Intraoral examination showed fair oral hygiene multiple fixed prosthodontic restorations and multiple carious teeth, multiple remaining roots and lipomatous tongue atrophy (Figure 1, Figure 2). The patient was referred to her neurologist to provide a detailed history about her medical condition and to coordinate with him regarding the steroid dose take to avoid any complication during patient management.

A consultation letter had been received from her neurologist explaining the patient medical condition which starts with difficulty in breathing, chewing and swallowing, there was no external ocular weakness, motor weakness, sphincter dysfunction, sensory deficits, muscle wasting and no signs of diplopia, dizziness, double vision, ptosis or any difficulty in maintaining balance. Patient underwent chest CT scan with contrast which show evidence of thymoma benign tumor of the thymus that has been the possible causes of MG. Diagnosis was confirmed with measuring serum level of acetylcholine receptor antibodies, later treatment plan had been formulated including drugs to control the condition and then thymectomy to avoid sever complication to the patient. Before proceeding to the dental management of the case; CBC and blood chemistry had been requested form the patient. Creatinine level was below normal (42.23 umol/l), with normal levels of albumin, urea, uric acid, AST, ALT, cholesterol, triglycerides and bilirubin. Patient had elevated WBC “12.52 x 10^9^/L” and Platelet “453 x 10^9^/L” with reduction in RBC “4.4 x 10^9^/L” and Eosinophil count “0.07”. Consultation with patient neurologist regarding the steroid dose to reduce the susceptibility of developing adrenal crisis.

## Discussion

Early involvement of areas innervated by the cranial nerves is commonly occur in MG disease. Patients’ exhibit ptosis, diplopia, difficulty in chewing or swallowing, respiratory challenges, limb weakness, or conjunction of these complications. In some patients, the disease remains limited to the eye muscles, but in most cases, it advances to other cranial nerve as well as to the shoulders and limbs. Diagnosis is mainly depending on a characteristic history of fluctuating diploma, ptosis, dysarthria and limb weakness [8]. Dysphagia, dyspnea and head droop may also be present. Muscles involved are fatigable and symptoms improve with rest [10]. However, the clinical presentation of this case was atypical, tongue atrophy and facial muscles weakness was only recorded in the absence of other clinical features of MG that may be misdiagnosed as facial palsy. Several reports had linked thymoma and MG, the patient’s genetic profile and the thymic ability to develop autoreactive T lymphocytes are the major reason to develop MG. MG has a genetic association to HLA-DR3 or ancestral haplotype 8.1 in early-onset MG with thymic hyperplasia and several weaker associations to polymorphisms in immunoregulatory genes such as FcγR, TNF-α/β, GM-phenotypes, CTLA-4 [11], HLA and PTPN22 * R620W [12].

The incidence of having a thymoma increases with the increase in number of polymorphisms in MG patient, indicating that thymoma MG is a polygenic disease and that thymoma patients with a particular genetic profile run higher risk of developing MG [12], in our case the thymoma that has been involved as the possible origin of the pathology of developing MG. The presentations of bulbar symptoms are well recognized in myasthenia gravis, but tongue atrophy is uncommon and usually present latter in course of the disease. There is only one case in the literature reported by Burch J *et al.* [13] similar to this report where the tongue atrophy is manifested first in MG in the absence of other clinical symptoms. Dental management of patients have MG presents a defiance to the dentist so, we need special management considerations to avoid a myasthenic crisis and modifying dental management to accommodate altered muscle strength [10]. Preoperative plasma exchange may be required for the patient with frequent myasthenia crises who is undergoing major oral surgery or in patients with high risk of exacerbation of myasthenic symptoms is expected. It is considered as a short term immunotherapy with temporary effect. Our case did not give a history of frequent crisis that there was no clear indication for plasmapheresis preoperatively [10].

The planning of dental treatment for MG patients necessitate specific contemplations, as well as advice and precautions with which all oral health care provider should be sufficiently aware of the disease symptoms and complications. A complete review of the patient’s symptoms and an assessment of speak and swallowing should be done before any treatment. It is recommended that multiple and short early morning appointments are preferable in all cases of MG, in order to reduce the development of cumulative muscle weakness and also to take privilege of the greater muscular strength which is maximum during morning hours [14]. For long procedure like endodontic treatment or extraction to achieve maximal strength of the oral and neck muscles we give the patient oral AChEs agents 1.5 hours should be administered before the dental treatment. One of the side effects of AChEs increased salivation, so it is impotent to use the rubber dam because the MG patient at the risk of aspiration of oral debris or saliva [15]. Prednisone and other immunosuppressive drugs such as azathioprine are commonly prescribed in the treatment of MG. Although these medication have great outcomes but, they may have an unfavorable effect specially on the efficiency of immune system with increased risk of opportunistic infections such as chronic mucocutaneous candidiasis, oral infections and delayed wound healing that make prophylactic antibiotic and antifungal therapy very important prior to dental procedures [16]. Ester-type local anesthetics, such as procaine, should be avoided in myasthenic patients as they hydrolyzed by plasma cholinesterase which are already deficient in MG patients. Lidocaine or mepivacaine which are amide-type local anesthetics, can be administered safely in these patients. Reports recommend using intraligamentary or intrapulpal local to reduce doses of the local anesthesia when possible [17, 18].

Following tooth extraction penicillin had been prescribed to reduce the susceptibility for opportunistic infection. Several studies revealed that using antibiotics should be used with caution, there are specific antibiotics that may cause muscle weakness due to their ability to produce partial neuromuscular blockade through inhibiting acetylcholine release from the presynaptic membrane. Aminoglycosides (gentamicin, streptomycin, amicacin, neomycin, kanamycin) must be avoided as they are known to cause clinically significant muscle weakness by blocking presynaptic voltage-activated calcium channels [19, 20].

## Conclusion

MG may be life threatening if a significant risk of aspiration develops, although this severity of illness is often associated with more serious complication involving the respiratory muscles which may require intubation and ventilatory support in hospitals. A continuous consultation between dental specialists and patient’s physician is very important prior to dental management, particularly if progression of symptoms is noted. Dentists should be familiar that alterations in facial expression, chewing and swallowing difficulty and weakness including dysarthria, dysnea, diplopia, dysphagia and tongue atrophy may be due to MG, and the condition is particularly critical if the patient reports ptosis, diplopia or if there is a background marked by thymoma or past MG.

## Competing interests

The authors declare no competing interests.

## References

[1] Souadjian JV, Enriquez P, Silverstein MN (1974). The spectrum of diseases associated with thymoma: coincidence or syndrome?. Arch Intern Med.

[2] Venuta F, Anile M, Diso D (2010). Thymoma and thymic carcinoma. Eur J Cardiothorac Surg.

[3] Detterbeck FC, Parsons AM (2004). Thymic tumors. Ann Thorac Surg.

[4] Cheney RT (2010). The biologic spectrum of thymic epithelial neoplasms: current status and future prospects. J Natl Compr Canc Netw.

[5] Romi F, Skeie GO, Aarli JA (2000). Muscle autoantibodies in subgroups of myasthenia gravis patients. J Neurol.

[6] Albert ML, Darnell RB (2004). Paraneoplastic neurological degenerations: keys to tumour immunity. Nat Rev Cancer.

[7] Romi F, Bø L, Skeie GO (2002). Titin and ryanodine receptor epitopes are expressed in cortical thymoma along with costimulatory molecules. J Neuroimmunol.

[8] Skeie GO, Bentsen PT, Freiburg A (1998). Cell-mediated immune response against titin in myasthenia gravis: evidence for the involvement of Th1 and Th2 cells. Scand J Immunol.

[9] Romi F, Skeie GO, Gilhus NE (2005). Striational antibodies in myasthenia gravis: reactivity and possible clinical significance. Arch Neurol.

[10] McGrogan A, Sneddon S, de Vries CS (2010). The incidence of myasthenia gravis: a systematic literature review. Neuroepidemiology.

[11] Chuang W-Y, Ströbel P, Gold R (2005). A CTLA4high genotype is associated with myasthenia gravis in thymoma patients. Ann Neurol.

[12] Amdahl C, Alseth EH, Gilhus NE (2007). Polygenic disease associations in thymomatous myasthenia gravis. Arch Neurol.

[13] Burch J, Warren-Gash C, Ingham V (2006). Myasthenia gravis-a rare presentation with tongue atrophy and fasciculation. Age Ageing.

[14] Rai B (2006). Myasthenia gravis: challenge to dental profession. Internet J Acad Phys Assist.

[15] Patil PM, Singh G, Patil SP (2012). Dentistry and the myasthenia gravis patient: a review of the current state of the art. Oral Surg Oral Med Oral Pathol Oral Radiol.

[16] Weksler B, Lu B (2014). Alterations of the immune system in thymic malignancies. J Thorac Oncol.

[17] Berrih-Aknin S, Le Panse R (2014). Myasthenia gravis: a comprehensive review of immune dysregulation and etiological mechanisms. J Autoimmun.

[18] Weijnen FG, van der Bilt A, Kuks JB (2002). Masticatory performance in patients with myasthenia gravis. Arch Oral Biol.

[19] Harnett MT, Chen W, Smith SM (2009). Calcium-sensing receptor: a high-affinity presynaptic target for aminoglycoside-induced weakness. Neuropharmacology.

[20] Jones SC, Sorbello A, Boucher RM (2011). Fluoroquinolone-associated myasthenia gravis exacerbation. Drug Saf.

